# Nonlinear Imaging Histopathology: A Pipeline to Correlate Gold-Standard Hematoxylin and Eosin Staining With Modern Nonlinear Microscopy

**DOI:** 10.1109/jstqe.2022.3233523

**Published:** 2023-01-02

**Authors:** Kayvan Forouhesh Tehrani, Jaena Park, Eric J. Chaney, Haohua Tu, Stephen A. Boppart

**Affiliations:** Beckman Institute for Advanced Science and Technology, University of Illinois Urbana-Champaign, Urbana, IL 61801-3028 USA; Beckman Institute for Advanced Science and Technology, University of Illinois Urbana-Champaign, Urbana, IL 61801-3028 USA, and also with the Department of Bioengineering, University of Illinois Urbana-Champaign, Urbana, IL 61801-3028 USA; Beckman Institute for Advanced Science and Technology, University of Illinois Urbana-Champaign, Urbana, IL 61801-3028 USA; Beckman Institute for Advanced Science and Technology, University of Illinois Urbana-Champaign, Urbana, IL 61801-3028 USA, and also with the Department of Electrical and Computer Engineering, University of Illinois Urbana-Champaign, Urbana, IL 61801-3028 USA; Beckman Institute for Advanced Science and Technology, Department of Electrical and Computer Engineering, Department of Bioengineering, Carle Illinois College of Medicine, and Cancer Center at Illinois, University of Illinois Urbana-Champaign, Urbana, IL 61801-3028 USA

**Keywords:** Histopathology, hematoxylin and eosin staining, label-free microscopy, nonlinear microscopy, multi-photon

## Abstract

Hematoxylin and eosin (H&E) staining, the century-old technique, has been the gold standard tool for pathologists to detect anomalies in tissues and diseases such as cancer. H&E staining is a cumbersome, time-consuming process that delays and wastes precious minutes during an intraoperative diagnosis. However, even in the modern era, real-time label-free imaging techniques such as simultaneous label-free autofluorescence multiharmonic (SLAM) microscopy have delivered several more layers of information to characterize a tissue with high precision. Still, they have yet to translate to the clinic. The slow translation rate can be attributed to the lack of direct comparisons between the old and new techniques. Our approach to solving this problem is to: 1) reduce dimensions by pre-sectioning the tissue in 500 μm slices, and 2) produce fiducial laser markings which appear in both SLAM and histological imaging. High peak-power femtosecond laser pulses enable ablation in a controlled and contained manner. We perform laser marking on a grid of points encompassing the SLAM region of interest. We optimize laser power, numerical aperture, and timing to produce axially extended marking, hence multilayered fiducial markers, with minimal damage to the surrounding tissues. We performed this co-registration over an area of 3 × 3 mm^2^ of freshly excised mouse kidney and intestine, followed by standard H&E staining. Reduced dimensionality and the use of laser markings provided a comparison of the old and new techniques, giving a wealth of correlative information and elevating the potential of translating nonlinear microscopy to the clinic for rapid pathological assessment.

## I. Introduction

THE most common histopathology stains used on thin sections of tissue specimens are hematoxylin and eosin (H&E) [1], which produce contrast in the cell nuclei and the extracellular matrix, respectively. H&E, which has been the standard method of histopathology for several decades [2], gives the “doctor’s doctor” (the pathologist) an understanding of the phenotype and density of cells, and the overall structure of the tissue. For instance, this information can be used to distinguish healthy from neoplastic samples when an abnormal proliferation of cells is observed or if the microstructure has been altered. Although histopathology with H&E staining is a powerful tool, it involves a time-consuming, laborious, and costly process [3], [4], often producing only a limited amount of information. In surgical oncology, for example, results often lead to compromised margins, the need for re-excision surgeries, and higher morbidity [5], [6]. On the other hand, modern optical imaging technologies have shown great promise to change the paradigm for speed, accuracy, and subjectivity of diagnosis by producing several more layers of label-free contrast and comprehensive information, examining tissue consistency, structure, and function in real-time, almost instantaneously [7], [8].

Advances in nonlinear optical microscopy have led to label-free imaging techniques that can produce high-resolution molecular, metabolic, and structural contrast. Linear [9], [10], [11] and nonlinear [12], [13] microscopy modalities have been used to express several different metabolic [14], [15], [16], structural [17], [18], [19], [20], [21], [22] and chemical [23], [24], [25] biomarkers. This wealth of information can be used not only for the diagnosis of excised biopsies [26] or intraoperative assessment [27], but also they can be used to assess the healing process [18], [28], [29].

Our lab has previously pioneered a multimodal microscopy technique, called Simultaneous Label-free Autofluorescence Multi-harmonic (SLAM) microscopy [13], which by programming of the spectrum and shaping of ultrafast pulses, can readily extract metabolic and structural information using autofluorescence signals from nicotinamide adenine dinucleotide (NADH), and flavin adenine dinucleotide (FAD) metabolic coenzymes, as well as purely structural information from Second and Third Harmonic Generation (SHG and THG) modalities, which respectively express information on fibrous structures (e.g., collagen, myosin), and optical heterogeneity (e.g., boundaries). The tailored ultrafast pulses of SLAM enable simultaneous 3-photon excitation of NADH and 2-photon excitation of FAD with both spatial and temporal co-registration using the same laser spectrum, enabling accurate redox ratio measurements reflective of metabolic processes [15].

These contrast mechanisms work best with freshly excised tissue, not requiring any chemical treatment of the tissue (e.g., fixation, paraffin embedding, sectioning into thin slices, staining, etc.), unlocking the potential for immediate imaging and diagnosis of a biopsy, even at the point-of-care or point-of-procedure. Conversely, H&E histology requires either snap-freezing or paraffin embedding of the tissue, sectioning into multiple thin (~5 μm) slices, followed by mounting on microscope slides and finally staining with markers of a limited number of cellular or structural features. This long and cumbersome procedure sometimes has to be performed while a cancer patient is under anesthesia undergoing a tumor resection surgery, delaying the initial diagnosis by the pathologist and the intraoperative procedure by the surgeon. The current standard of care relies on technologies that have been proven and demonstrated for many years, although they might be time-consuming, or limited in their aspects of specific information. With the aging of the U.S. population and the increased demand for high-quality healthcare, there is a solid need to renovate these disease diagnosis workflows by demonstrating and proving the span of new diagnostic-aiding information that microscopy can add to the pathologists’ toolbox. This can only become possible by extensive correlative histology–microscopy analysis, which is non-trivial since meticulous and precise technique and effort is needed to match a thin optical section with its physical counterpart within a large tissue after tissue altering chemical treatments for histology. Previous efforts by us and others very often involve best-efforts and trial and error in finding a slice that closely matches the imaging field of view (FOV) [10], [30]. In this paper, we present a workflow that enables close comparison and correlation of SLAM microscopy and paraffin-embedded H&E histology.

Our approach for correlative imaging of the two technologies consists of first imaging a sample with the SLAM microscope, then processing the tissue for H&E staining while maintaining reference points or fiducial markers, followed by white light imaging of the stained slides. Because the sample is often bulky (tens of millimeters cubed), and the microscope can image very thin slices of this volume, coordination of these two efforts must be precisely concerted to achieve the same FOV in both methods. Other than matching the coordinates in x, y, and z, the yaw and pitch of oblique angles have to match, while the sample is processed and embedded in paraffin, making it a non-trivial task. Our method for correlation of the two methods is a four-prong approach: 1) reduce the dimensions, 2) add a reference plane, 3) add laser-induced fiducial markings that appear on both imaging methods, and 4) laser alignment of the microtome vs. the reference plane. In the first step, the tissue is pre-sectioned to thinner slices in the 200-500 micron range. Then the slice is placed on a coverglass which acts as the reference plane and stays on throughout the process (step 2). Since the coverglass is maintained in parallel to the SLAM imaging plane, it can be used as the reference point. For samples with indistinct shapes, laser markings are used to make a guide between the two methods (step 3). Finally, laser alignment is performed to ensure that the microtome sectioning plane is matched with the imaging plane. Following these steps enables a close match between label-free SLAM images of fresh tissues and images of H&E-stained tissue sections. We present a close correlation of slices of mouse intestine and kidney with and without laser marking, respectively. We show that the vibratome sectioning and embedding of the tissue in agarose gel do not affect the metabolism of the tissue and preserve the tissue for a longer time.

## II. Methods

### A. Tissue Preparation

**Fig. 1.**
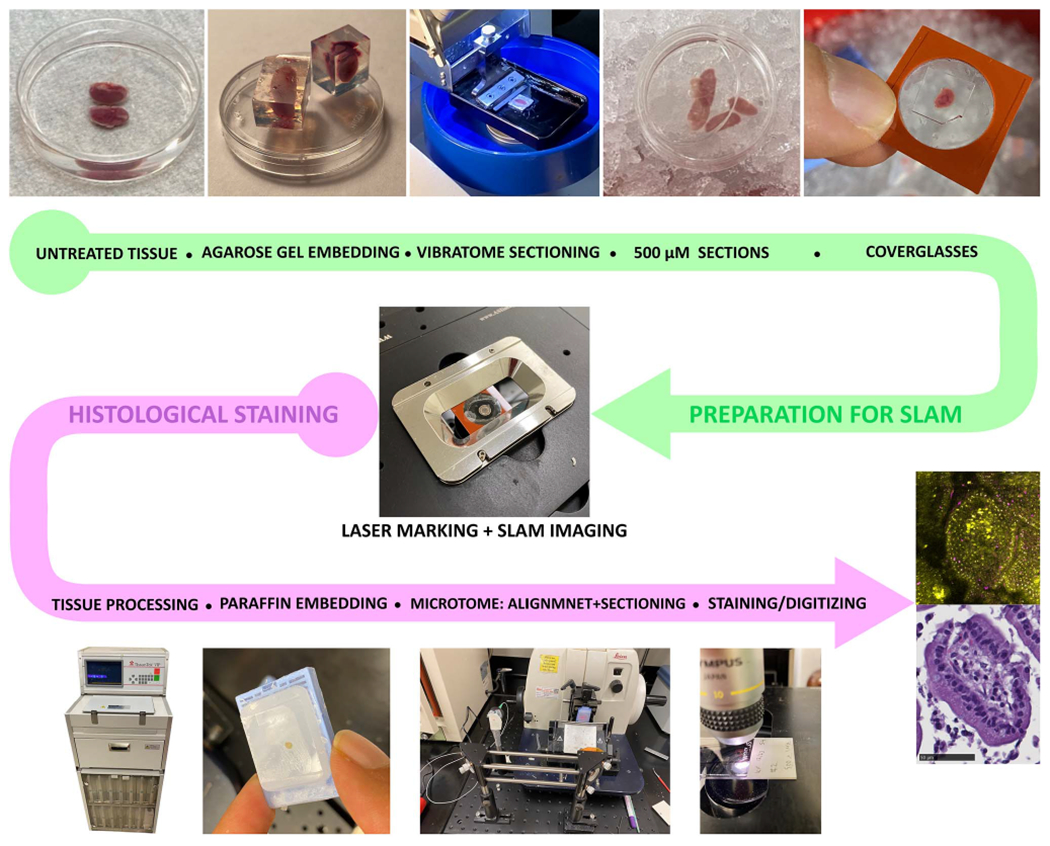
Pipeline from freshly excised tissue to correlated SLAM-H&E images. The first path (green) in the pipeline prepares the tissue for SLAM imaging by reducing the dimensions (vibratome sectioning) and adding a reference point (coverglass). In the second path (purple) of the pipeline, the imaged sample is prepared for H&E stained imaging, while the reference point is maintained and laser-aligned to match the imaging and sectioning planes. Finally, the resulting images from both modalities are put together for comparison.

The pipeline (Fig. 1) accepts a freshly excised tissue specimen and embeds it in 6% low-melting point (LMP) agarose (Fisher scientific 50-136-8026) gel in phosphate-buffered saline (PBS) for vibratome sectioning. The gelling temperature of LMP agarose is ~30 °C, lower than the body temperature, thus minimally affecting the metabolism of the tissue. The temperature of the gel is measured after melting, and when it reaches 37 °C the tissue is added to the gel and then placed on ice for faster gelling. The tissue is subsequently sectioned into 500 μm slices using a vibratome. The slices are then sandwiched between a coverglass and a slide using a silicone press-to-seal spacer (EMS 70336-61). The resulting fresh mounted tissue slices are then imaged on the SLAM microscope. It must be noted that the aforementioned sectioning process is only for the purpose of this correlation pipeline, and it is not a regular preparation step for SLAM imaging. After imaging, the tissue and the coverglass are placed in a tissue processing cassette, where layers of foam pads are placed on top of the tissue as extra support to maintain its position. The cassette is initially placed in formalin and then in the tissue processor as per the standard for histology. Following this, the tissue, still attached to the coverglass, undergoes paraffin embedding. The tissue and coverglass, which are in molten paraffin, are placed in a mold and cooled to set, followed by the removal of any excess paraffin in front of the glass using a razor blade. The paraffin-blocked tissue is then placed in the microtome, where the pitch and yaw are corrected using the laser alignment system. Finally, the paraffin-embedded tissue is sectioned into 5 *μ*m sections. These sections are deparaffinized (10 minutes Xylene, 3 minutes 100% EtOH, 3 minutes 95% EtOH, 3 minutes 70% EtOH, 3 minutes 50% EtOH, 1 minute water wash) and stained with H&E (3 minutes Hematoxylin: Fisher Scientific 23-245677, 10 minutes wash with water, 30 second Eosin-Y: Fisher 314-630, 15 seconds 50% EtOH, 1 minute 70% EtOH, 1 minute 90% EtOH, 2 minutes 100% EtOH, 4 minutes Xylene) and the whole slides are digitized in a slide scanner (Nanozoomer, Hamamatsu).

### B. Laser Alignment

**Fig. 2.**
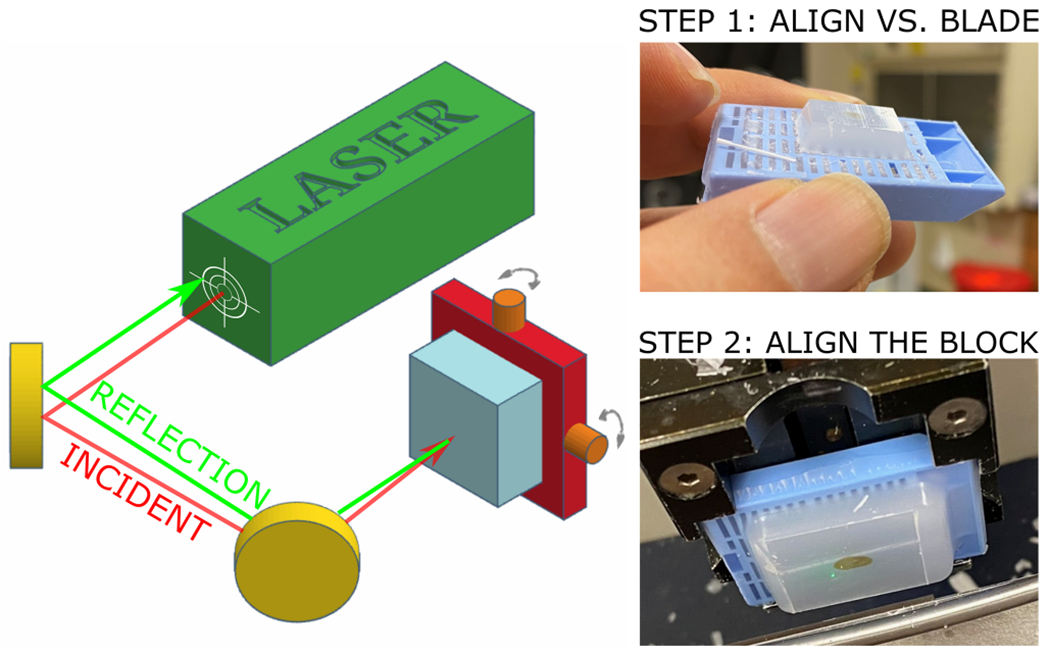
Microtome alignment. This process consists of two steps 1) aligning the laser steering versus the blade, using a finely sliced and reflective surface of paraffin, and 2) align the sample using the reflection from the surface of the coverglass.

**Fig. 3.**
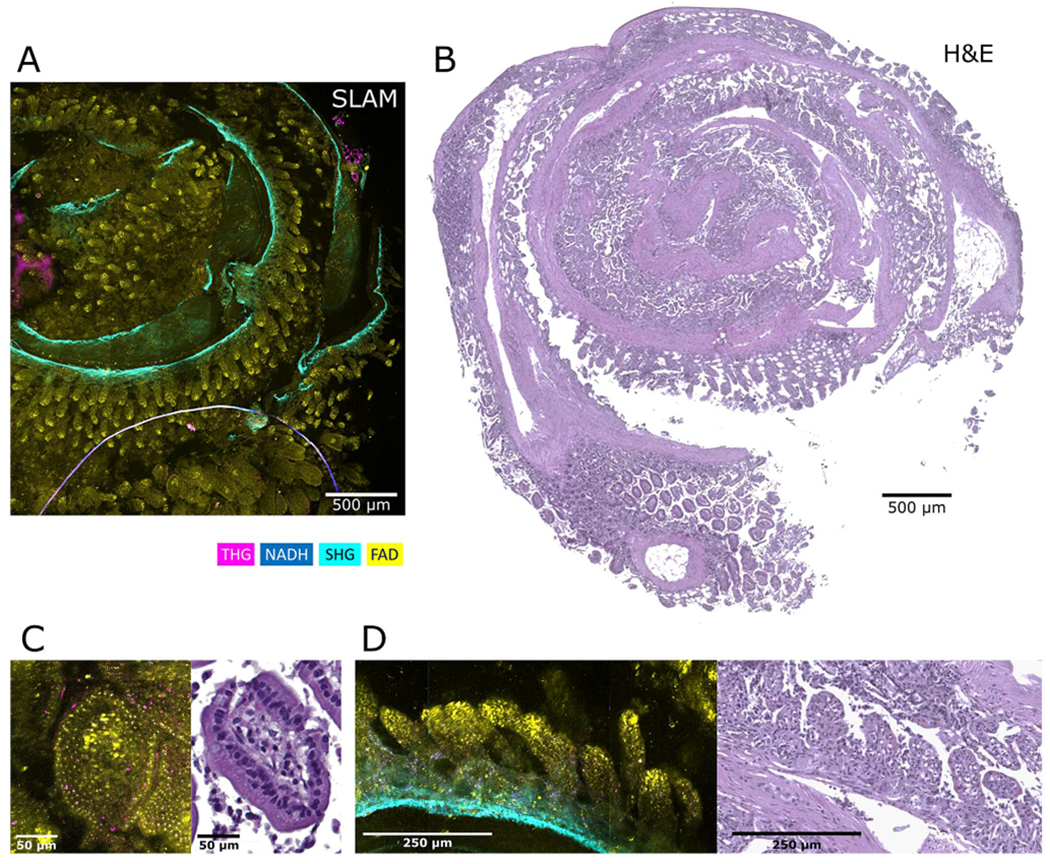
Correlative imaging of tissues containing distinct features. (A) Mosaic SLAM image of a rolled mouse small intestine, including the ileum and a small portion of the jejunum. (B) Histological staining of the section shown in (A). (C) Zoomed-in images of a crypt containing Paneth cells characterized by their granular structure. (D) Villi from the ileum.

The laser-aligned microtome system consists of a continuous wave laser (635 nm, Thorlabs HLS635), two mirrors, and the microtome (Fig. 2). The goal of this process is to make sure that the blade is fully parallel to the imaging plane within the sample, as determined by the coverglass orientation. Hence, the resulting sectioning plane will be in the same orientation as the imaging plane. Step one of the process is to align the laser beam with the blade, ensuring the beam is perpendicular to the blade plane. To do this, a paraffin block is finely (1 μm) sectioned to make a reflective surface, then without changing the yaw and pitch on the microtome, the two mirrors are adjusted to guide the beam back to its source, as shown in Fig. 3. Once the system is aligned, the reflecting paraffin block is exchanged with the sample block containing the reflecting coverglass. In step two of the process, the reflection from the surface of the coverglass on the paraffin block is used, and the yaw and pitch of the microtome are fine tuned to guide the laser beam reflection back to the source.

### C. Laser Marking

A separate optical setup was used for the generation of laser-induced fiducial markers. This system includes a water-immersion objective lens (Olympus LUMFLN60XW), for which the back aperture of the objective lens is underfilled to ~60% to reduce the numerical aperture, hence extending the tissue marking depth. A fiber laser source (Satsuma, Amplitude lasers) with ~370 fs pulses at a repetition rate of 1 MHz, and average power of 400 mW was used. The laser was incident at each spot for 500 ms on a 5 × 5 grid pattern with 500 *μ*m intervals.

### D. SLAM Microscopy

The SLAM microscope has previously been demonstrated and explained in several publications [13], [31], [32], [33], [34], [35], [36]. Briefly, this microscope employs a fiber laser with a center wavelength of 1030 nm, 20 MHz pulse repetition rate, and 378 fs pulses (Satsuma, Amplitude lasers). The laser is coupled into a photonic crystal fiber (NKT LMA-PM-15) to produce a supercontinuum of light which is then sent to a spatial light modulator based pulse compressor (MIIPSbox640, Biophotonics solutions) to compress the pulse duration down to 50 fs. The microscope uses two conjugated galvanometer mirrors for scanning, an Olympus objective lens (XLPLN25XWMP2), and four photon-counting photomultiplier tubes with the following filters (center wavelength / bandwidth): 370/10 nm, 450/60 nm, 555/30 nm, 610/60 nm, used for collection of THG, NADH, SHG, FAD signals, respectively. The average power at the sample was 14 mW and the pixel dwell time was 22 *μ*s. Individual images have a pixel size of 500 nm over a total of 900 × 900 pixels, requiring ~18 s acquisition time.

## III. Results and Discusion

### A. Fiducial-Free Correlation

The first example of correlative microscopy we present here is a fiducial-marker-free sample of a mouse small intestine that has been rolled together (Fig. 3). since this sample has distinct features, similar features can be found effortlessly. For this sample, the agarose gel was also maintained during the process, protecting the integrity of the sample. A comparison of SLAM and H&E images (Fig. 3(C), (D)) shows the additional information available within the SLAM image compared to H&E. For instance, the SLAM image illuminates the granular structure in the cells, a hallmark of the Paneth cells [37], making detection of the cell type relatively easy. In contrast, in the H&E image only the nuclei are demonstrated, with no specific distinction of the cellular features. In the villi of the ileum, while the H&E image shows the density of cells and the overall structure of the villi, the SLAM image shows not only the granular structures but also the external muscular layer, and the fibrillar collagen outer layer [38] through SHG contrast. The THG signal, in addition to the granular structure, produces contrast in the superficial epithelial layer of the villi. Moreover, the ratio of the two autofluorescence biomarkers (NADH, blue and FAD, yellow) superimposed, highlights the metabolic signature of the structures, essentially the different shades of green in the image tells us about the metabolic state of the tissue (yellow being healthiest, greener shows reduced metabolism). The comparison presented here may not be precisely one-to-one (pixel-wise comparison), due to the changes that occur during the imaging and the tissue processing. That is because a tissue is a dynamic environment, many chemical and physical interactions occur during the imaging, and post-processing can trigger mechanistic perturbation of the tissue. The illumination with the laser light - even though under the damage threshold, and later treatment with many chemicals for fixation and paraffin can make the shapes deviate from their original state. Nonetheless, with this correlative effort, the imaging plane has been preserved and perdured throughout the whole pipeline, enabling the comparison of H&E images with their corresponding SLAM contrast mechanisms and paving the way for further translation of histopathology to modern nonlinear microscopy.

### B. Laser Marked Fiducials

**Fig. 4.**
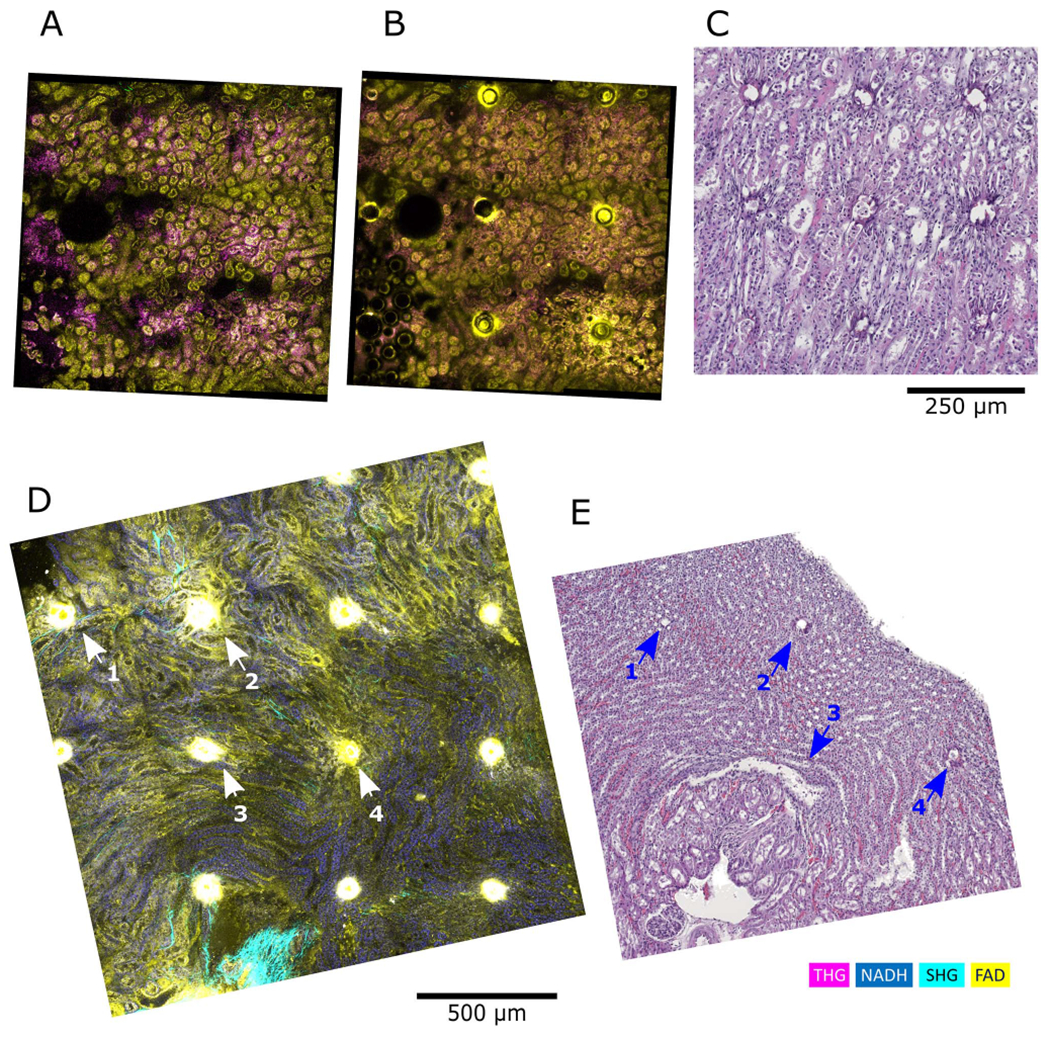
Laser-marking as fiducial markers for correlation. Femtosecond laser with ~370 fs pulses at a repetition rate of 1 MHz, and average power of 400 mW was shone for 500 ms on each spot. A rabbit kidney biopsy specimen imaged before and after laser marking is shown in (A) and (B), respectively. The corresponding histological staining of the same tissue is shown in (C). SLAM image of a mouse kidney slice with laser marking and its corresponding H&E-stained image are shown in (D) and (E), respectively.

However, samples that do not have distinct features require additional fiducial markings, enabling tracking of the same locations between SLAM and H&E. Tissues like kidney consist of multiple tubule structures in many orientations, and little to no uniquely distinctive features, making it challenging to co-register a similar FOV without fiducial markers. Here we present two examples of correlative microscopy using laser-induced fiducial markers (Fig. 4). In Fig. 4(A), (C), a rabbit kidney is shown before and after laser marking, along with its corresponding H&E image. This example shows the markers are clearly preserved between the two modalities, and several structures can be distinguished between these images. However, in this initial experiment, we realized that the laser markings might have adverse effects on tissue health; therefore, we optimized the laser marking protocol by adjusting spot distances, laser power, repetition rate, and duration to make sure there is no observable damage outside of the confined region of the spot, as presented in Fig. 4(D), (E).

#### A. Characterization of Laser-Induced Fiducial Marks

**Fig. 5.**
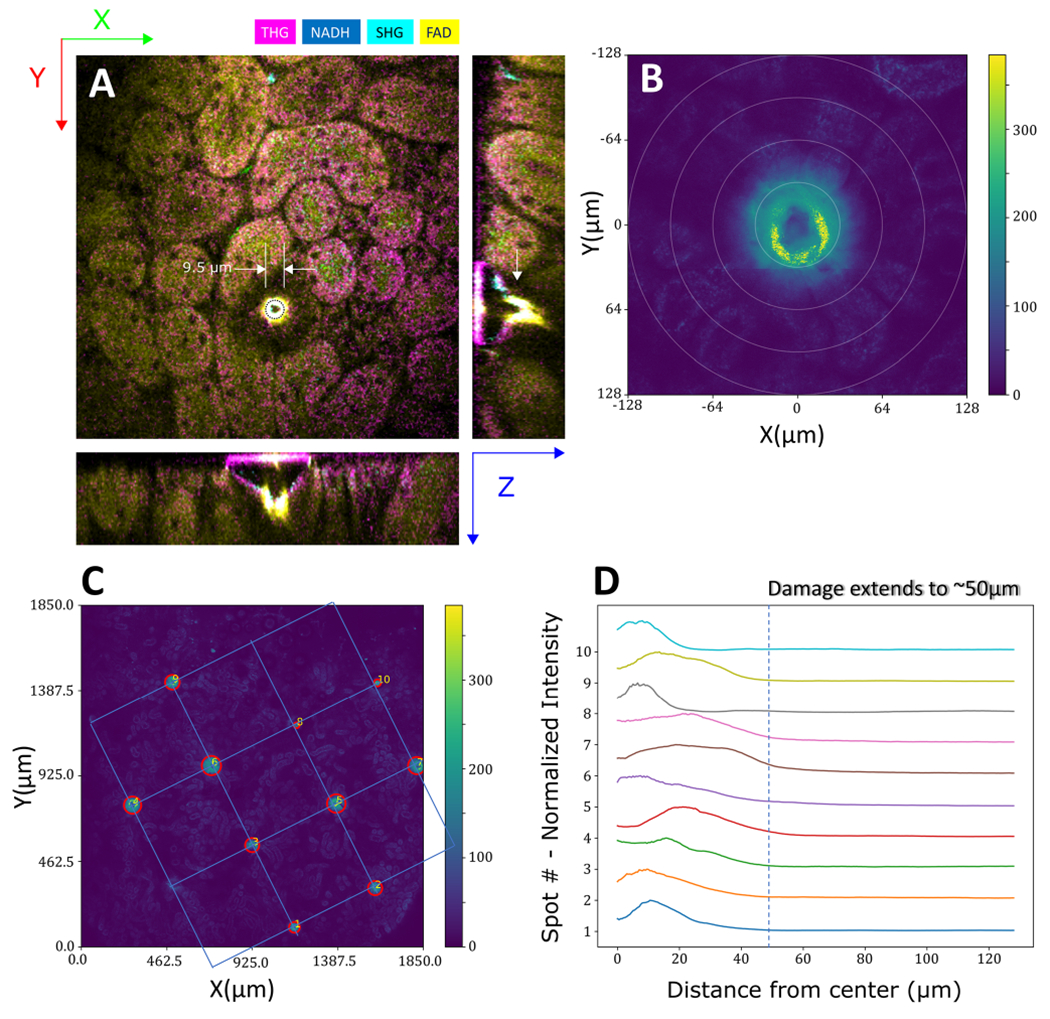
Laser-marking characterization. (A) A laser-induced mark along with its orthogonal lateral cross-sections. (B) Zoomed-in view of 2-photon autofluorescence signal from a single laser-induced marking, with the circles used for damage distance threshold analysis. (C) Positions of 2PAF (FAD) signal from multiple ablations. (D) Traces of the ablation spots from panel (C). Each point at distance x shown in (D) is an average of a circle with radius x, as shown in (B).

Although the laser markings show that the correlation between the two imaging methods is possible, it is important to understand the physical qualities of the marks and the health and integrity of the tissue sample after the laser exposure. Therefore, we quantified the laser-induced spots by measuring their diameter and depth, as shown in Fig. 5(A). The diameter of the marks was, on average, 36.4 ± 10.2 *μ*m (10 markings), with a depth of >50 *μ*m. To characterize the laser damage, the FAD (2-photon) autofluorescence signal of the marked region was collected and then averaged on rings originating from the center of the mark (Fig. 5(B), (D)). A characteristic of laser damage is raised signal levels in all channels due to laser-induced plasma generation, which can be inferred from the traces that the damage extends to a maximum of 50 *μ*m from the center. Also comparing the ratios of NADH and FAD signals (redox), shows no significant change outside of the laser marking area. Given that the spacing between the marks is 500 *μ*m, there exists substantial space for metabolic and structural analysis of the tissue through SLAM images, and its correlation with their corresponding H&E images.

#### B. Characterization of Imaging Following Gel Embedding

**Fig. 6.**
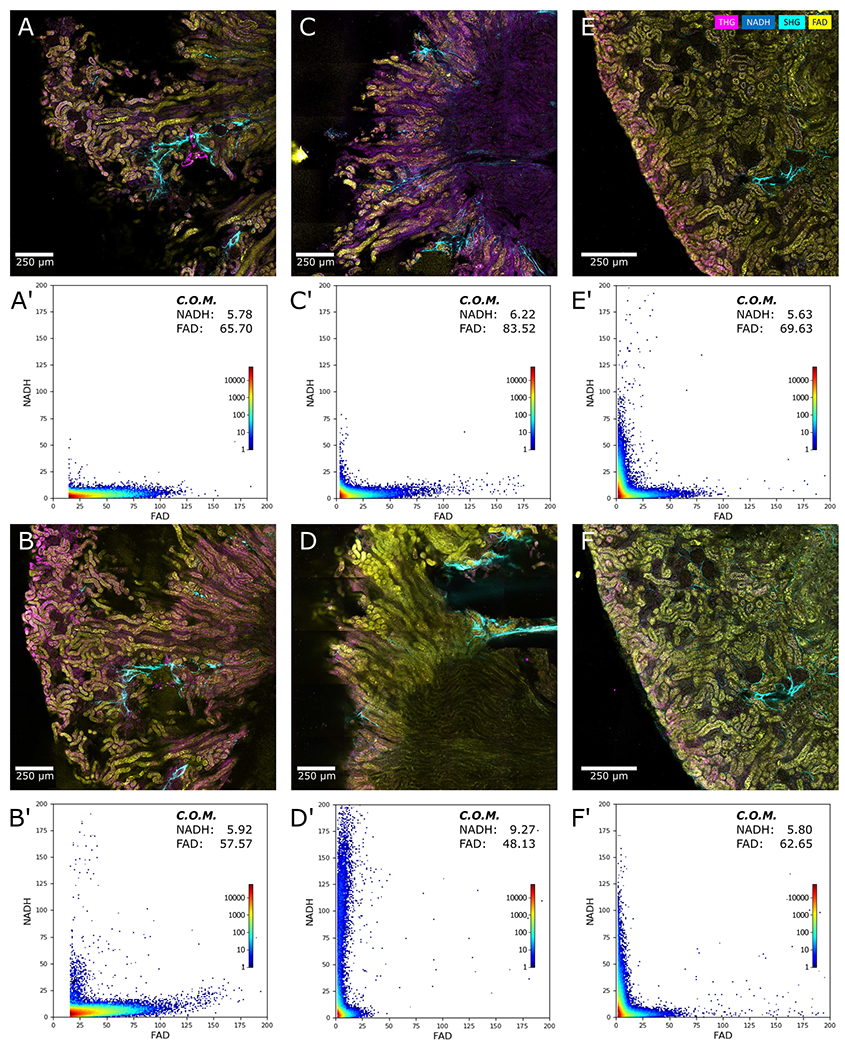
Comparison of tissue preservation with and without agarose gel embedding. Fresh mouse kidney samples were imaged immediately (A & C) without treatment. A second kidney sample was embedded in agarose gel, sliced, and sandwiched between two glasses, shown in (E). The samples shown in (A) and (E) were kept on ice for one hour, and (C) was placed in formalin at room temperature for 1 hour. These samples were imaged again after 1 hour, shown in (B, D, F) respectively. The corresponding graphs of NADH and FAD signals for each image are shown in their respective prime-marked panels (A’-F’). The center of mass (COM) for each graph is calculated and used to compare the metabolic changes quantitatively.

We also investigated whether embedding of the tissue in agarose gel affects the health and integrity of the sample. We excised the kidneys of a mouse, transversally sectioned them in half and imaged them at 0-hour and 1- hour timepoints under 3 conditions: i) unprocessed tissue kept on ice (Fig. 6(A), (B)), ii) unprocessed tissue placed in formalin (Fig. 6(C), (D)), and iii) gel-embedded coverglass-slide mounted tissue slice kept on ice (Fig. 6(E), (F)). To assess tissue health, we plotted graphs of NADH vs. FAD autofluorescence signals for each image and showed these respective panels marked prime (‘). To better quantify the tissue health status, we calculated the center of mass (COM) for each graph based on the contributing (non-zero) data points. As expected, in the first case, we see a slight shift of the COM, with the FAD signal reducing and NADH increasing due to reduced metabolism over time. In the case of the tissue treated with formalin at room temperature, the COM shifted significantly, with much-reduced FAD and increased NADH levels. However, in the case of the coverglass-slide mounted tissue, the COM shifted only slightly. It should also be noted that in all cases, we attempted to go back to the same FOV after 1 hour, but the untreated tissues had changed structurally and metabolically within this time frame, and it was impossible to capture the exact same FOV. However, in the case of the mounted tissue specimen, we could easily go back to the same FOV. This experiment highlights that the proposed process and pipeline, including agarose embedding and vibratome sectioning, not only does not damage the tissue, but also helps preserve the tissue architecture, which subsequently helps facilitate finding the same FOV for longitudinal imaging.

## IV. Conclusion

In conclusion, we presented a pipeline that enables correlative microscopy of conventional histopathology imaging with modern-day nonlinear optical imaging (SLAM microscopy). Label-free SLAM imaging allows for capturing several orthogonal and spatially and temporally co-registered layers of information instantaneously and noninvasively without needing laborious and time-consuming staining, as in conventional histopathology methods. SLAM microscopy can simultaneously acquire endogenous molecular signals from a wide array of cellular and extracellular components from both *in vivo* or *ex vivo* tissues, including diverse cells, vesicles, adipocytes, fibers, vessels, membranes, neural structures, and epithelial formations. Our presented pipeline enabled finding similar FOVs by reducing the dimensions of the tissue and adding laser-induced fiducial markers that stay with the sample and appears in both imaging methods, helping to correlate the FOVs. When the sample consists of distinct landmarks, dimension reduction and the use of a coverglass as a reference plane help with correlating the two modalities; however, with more homogenous samples, it is necessary to add the additional laser-induced markings to facilitate locating and aligning the same FOV. We successfully performed the placement of laser-induced fiducial markers and optimized laser parameters to minimize collateral damage to the sample. We also examined the effect of agarose gel embedding and found that not only does it not significantly alter the sample, but it also preserves the sample, and helps with finding the same FOV in longitudinal studies. We would also like to emphasize that although the comparisons between the two modalities are not precisely one-to-one on the micron-scale due to the complex tissue deformations that occur in both the fresh and the processed specimens, this pipeline still produces a wealth of correlative information that can pave the way for pathologists to augment and potentially even replace the current gold-standard histopathology technique with modern day optical microscopy. We will continue this work by applying this method to a larger scale collaborative effort with pathologists, on cancer or other disease specimen, to further show the potential of nonlinear and correlative microscopy to the scientific community.

## References

[1] King DF and King LAC, “A brief historical note on staining by hematoxylin and eosin,” Amer. J. Dermatopathol, vol. 8, no. 2, 1986, Art. no. 168. [Online]. Available: https://journals.lww.com/amjdermatopathology/Fulltext/1986/04000/A_Brief_Historical_Note_on_Staining_by_Hematoxylin.13.aspx 10.1097/00000372-198604000-00013 2424331

[2] Tadrous PJ, Diagnostic Criteria Handbook in Histopathology: A Surgical Pathology Vade Mecum. Hoboken, NJ, USA: Wiley, 2008.

[3] Buesa RJ, “Productivity standards for histology laboratories,” Ann. Diagn. Pathol, vol. 14, no. 2, pp. 107–124, Apr. 2010, doi: 10.1016/j.anndiagpath.2009.12.005. 20227016

[4] Buesa RJ, “Staffing benchmarks for histology laboratories,” Ann. Diagn. Pathol, vol. 14, no. 3, pp. 182–193, 2010, doi: 10.1016/j.anndiagpath.2010.02.001. 20471564

[5] Sabel MS , “Residual disease after re-excision lumpectomy for close margins,” J. Surg. Oncol, vol. 99, no. 2, pp. 99–103, Feb. 2009, doi: 10.1002/jso.21215. 19065638

[6] Kobbermann A. , “Impact of routine cavity shave margins on breast cancer re-excision rates,” Ann. Surg. Oncol, vol. 18, no. 5, pp. 1349–1355, May. 2011, doi: 10.1245/s10434-010-1420-6. 21046260

[7] Cahill LC , “Nonlinear microscopy for detection of prostate cancer: Analysis of sensitivity and specificity in radical prostatectomies,” Modern Pathol, vol. 33, no. 5, pp. 916–923, May 2020, doi: 10.1038/s41379-019-0408-4. PMC7195230 31745288

[8] You S. , “Label-free deep profiling of the tumor microenvironment,” Cancer Res, vol. 81, no. 9, pp. 2534–2544, May 2021, doi: 10.1158/0008-5472.Can-20-3124. 33741692 PMC8137645

[9] Wang J, Chaney EJ, Aksamitiene E, Marjanovic M, and Boppart SA, “Compressive sensing for polarization sensitive optical coherence tomography,” J. Phys.D: Appl. Phys, vol. 54, no. 29, May 2021, Art. no. 294005, doi: 10.1088/1361-6463/abf958. PMC10786634 38222471

[10] Masters BR, “Correlation of histology and linear and nonlinear microscopy of the living human cornea,” J. Biophoton, vol. 2, no. 3, pp. 127–139, Mar. 2009, doi: 10.1002/jbio.200810039. 19343693

[11] Mittal S , “Simultaneous cancer and tumor microenvironment subtyping using confocal infrared microscopy for all-digital molecular histopathology,” Proc. Nat. Acad. Sci, vol. 115, no. 25, pp. E5651–E5660, Jun. 2018, doi: 10.1073/pnas.1719551115. 29866827 PMC6016804

[12] Tu H. , “Stain-free histopathology by programmable supercontinuum pulses,” Nature Photon, vol. 10, no. 8, pp. 534–540, Aug. 2016, doi: 10.1038/nphoton.2016.94. PMC5031149 27668009

[13] You S. , “Intravital imaging by simultaneous label-free autofluorescence-multiharmonic microscopy,” Nature Commun, vol. 9, no. 1, May 2018, Art. no. 2125, doi: 10.1038/s41467-018-04470-8. PMC5974075 29844371

[14] Rothstein EC, Carroll S, Combs CA, Jobsis PD, and Balaban RS, “Skeletal muscle NAD(P)H two-photon fluorescence microscopy in vivo: Topology and optical inner filters,” Biophys. J, vol. 88, no. 3, pp. 2165–2176, 2005, doi: 10.1529/biophysj.104.053165. 15596503 PMC1305268

[15] Skala MC , “In vivo multi-photon microscopy of NADH and FAD redox states, fluorescence lifetimes, and cellular morphology in precancerous epithelia,” in Proc. Nat. Acad. Sci, vol. 104, no. 49, pp. 19494–19499, 2007, doi: 10.1073/pnas.0708425104. 18042710 PMC2148317

[16] Tehrani KF, Pendleton EG, Southern WM, Call JA, and Mortensen LJ, “Two-photon deep-tissue spatially resolved mitochondrial imaging using membrane potential fluorescence fluctuations,” Biomed. Opt. Exp, vol. 9, no. 1, pp. 254–259, Jan. 2018, doi: 10.1364/BOE.9.000254. PMC5772580 29359101

[17] Tehrani KF , “Five-dimensional two-photon volumetric microscopy of in-vivo dynamic activities using liquid lens remote focusing,” Biomed. Opt. Exp, vol. 10, no. 7, pp. 3591–3604, 2019. [Online]. Available: http://www.osapublishing.org/boe/abstract.cfm?URI=boe-10-7-3591 10.1364/BOE.10.003591 PMC6640832 31360606

[18] Forouhesh Tehrani K, Pendleton EG, Southern WM, Call JA, and Mortensen LJ, “Spatial frequency metrics for analysis of microscopic images of musculoskeletal tissues,” Connective Tissue Res, vol. 62, no. 1, pp. 4–14, 2021, doi: 10.1080/03008207.2020.1828381. PMC7718369 33028134

[19] Chen X, Nadiarynkh O, Plotnikov S, and Campagnola PJ, “Second harmonic generation microscopy for quantitative analysis of collagen fibrillar structure,” Nature Protoc, vol. 7, pp. 654–669, 2012, doi: 10.1038/nprot.2012.009. 22402635 PMC4337962

[20] Chen X, Raggio C, and Campagnola PJ, “Second-harmonic generation circular dichroism studies of osteogenesis imperfecta,” Opt. Lett, vol. 37, no. 18, pp. 3837–3839, 2012, doi: 10.1364/OL.37.003837. 23041876 PMC4337953

[21] Campbell KR , “3D second harmonic generation imaging tomography by multi-view excitation,” Optica, vol. 4, no. 10, pp. 1171–1179, 2017, doi: 10.1364/OPTICA.4.001171. 29541654 PMC5847324

[22] Tehrani KF, Kner P, and Mortensen LJ, “Characterization of wavefront errors in mouse cranial bone using second-harmonic generation,” J. Biomed. Opt, vol. 22, no. 3, 2017, Art. no. 036012, doi: 10.1117/1.JBO.22.3.036012. 28323304

[23] Sarri B. , “Fast stimulated Raman and second harmonic generation imaging for intraoperative gastro-intestinal cancer detection,” Sci. Rep, vol. 9, no. 1, 2019, Art. no. 10052, doi: 10.1038/s41598-019-46489-x. PMC6624250 31296917

[24] Saar BG, Johnston RS, Freudiger CW, Xie XS, and Seibel EJ, “Coherent Raman scanning fiber endoscopy,” Opt. Lett, vol. 36, no. 13, pp. 2396–2398, 2011, doi: 10.1364/OL.36.002396. 21725423 PMC3164497

[25] Cadena A. D. l. , “Broadband stimulated Raman imaging based on multi-channel lock-in detection for spectral histopathology,” APL Photon. vol. 7, no. 7, 2022, Art. no. 076104, doi: 10.1063/5.0093946.

[26] Cahill LC , “Rapid virtual hematoxylin and eosin histology of breast tissue specimens using a compact fluorescence nonlinear microscope,” Lab. Investigation, vol. 98, no. 1, pp. 150–160, 2018, doi: 10.1038/labinvest.2017.116. PMC5752596 29131161

[27] Yang L. , “Label-freemultimodal nonlinear optical imaging of needle biopsy cores for intraoperative cancer diagnosis,” J. Biomed. Opt, vol. 27, no. 5, 2022, Art. no. 056504, doi: 10.1117/1.JBO.27.5.056504. PMC9142840 35643823

[28] Chamberlain CS, Crowley EM, Kobayashi H, Eliceiri KW, and Vanderby R, “Quantification of collagen organization and extracellular matrix factors within the healing ligament,” Microsc. Microanal, vol. 17, no. 5, pp. 779–787, 2011, doi: 10.1017/S1431927611011925. 21910939 PMC3263369

[29] Southern WM , “PGC-1*α* overexpression partially rescues impaired oxidative and contractile pathophysiology following volumetric muscle loss injury,” Sci. Rep, vol. 9, no. 1, 2019, Art. no. 4079, doi: 10.1038/s41598-019-40606-6. PMC6411870 30858541

[30] van Huizen LMG , “Compact portable multi-photon microscopy reveals histopathological hallmarks of unprocessed lung tumor tissue in real time,” Transl. Biophoton, vol. 2, no. 4, 2020, Art. no. e202000009, doi: 10.1002/tbio.202000009. PMC8311669 34341777

[31] Park J. , “Label-free optical redox ratio from urinary extracellular vesicles as a screening biomarker for bladder cancer,” Amer. J.Cancer Res, vol. 12, no. 5, pp. 2068–2083, 2022. [Online]. Available: https://www.ncbi.nlm.nih.gov/pmc/articles/PMC9185616/pdf/ajcr0012-2068.pdf 35693090 PMC9185616

[32] You S. , “Label-free visualization and characterization of extracellular vesicles in breast cancer,” in Proc. Nat. Acad. Sci, vol. 116, no. 48, 2019, pp. 24012–24018, doi: 10.1073/pnas.1909243116. 31732668 PMC6883786

[33] You S. , “Real-time intraoperative diagnosis by deep neural network driven multi-photon virtual histology,” NPJ Precis. Oncol, vol. 3, no. 1, 2019, Art. no. 33, doi: 10.1038/s41698-019-0104-3. PMC6917773 31872065

[34] Lee JH , “Simultaneous label-free autofluorescence and multiharmonic imaging reveals in vivo structural and metabolic changes in murine skin,” Biomed. Opt. Exp, vol. 10, no. 10, pp. 5431–5444, 2019, doi: 10.1364/BOE.10.005431. PMC6788598 31646056

[35] You S. , “Slide-free virtual histochemistry (Part I): Development via nonlinear optics,” Biomed. Opt. Exp, vol. 9, no. 11, pp. 5240–5252, 2018, doi: 10.1364/BOE.9.005240. PMC6238939 30460125

[36] You S. , “Slide-free virtual histochemistry (Part II): Detection of field cancerization,” Biomed. Opt. Exp, vol. 9, no. 11, pp. 5253–5268, 2018, doi: 10.1364/BOE.9.005253. PMC6238923 30460126

[37] Jang WH , “Two-photon microscopy of Paneth cells in the small intestine of live mice,” Sci. Rep, vol. 8, no. 1, 2018, Art. no. 14174, doi: 10.1038/s41598-018-32640-7. PMC6155010 30242205

[38] Parker GA , “Histopathological features of the development of intestine and mesenteric lymph node injury in a nonhuman primate model of partial-body irradiation with minimal bone marrow sparing,” Health Phys, vol. 116, no. 3, pp. 426–446, Mar. 2019, doi: 10.1097/hp.0000000000000932. 30624355 PMC6362996

